# Anhedonia severity mediates the relationship between attentional networks recruitment and emotional blunting during music listening

**DOI:** 10.1038/s41598-024-70293-x

**Published:** 2024-08-29

**Authors:** Marie-Stephanie Cahart, Vincent Giampietro, Laura Naysmith, Mathilde Muraz, Fernando Zelaya, Steven C. R. Williams, Owen O’Daly

**Affiliations:** Neuroimaging Department, Institute of Psychiatry, Psychology and Neuroscience, Kings College London, 16 De Crespigny Park, London, SE5 8AB UK

**Keywords:** Neuroscience, Psychology

## Abstract

Emotion studies have commonly reported impaired emotional processing in individuals with heightened anhedonic depressive symptoms, as typically measured by collecting single subjective ratings for a given emotional cue. However, the interindividual variation in moment-to-moment emotional reactivity, and associated time-varying brain networks recruitment as emotions are unfolding, remains unclear. In this study, we filled this gap by using the unique temporal characteristics of music to investigate behavioural and brain network dynamics as a function of anhedonic depressive symptoms severity. Thirty-one neurotypical participants aged 18–30 years completed anhedonic depression questionnaires and then continuously rated happy, neutral and sad pieces of music whilst undergoing MRI scanning. Using a unique combination of dynamic approaches to behavioural (i.e., emotion dynamics) and fMRI (i.e., leading eigenvector dynamics analysis; LEiDA) data analysis, we found that participants higher in anhedonic depressive symptoms exhibited increased recruitment of attentional networks and blunted emotional response to both happy and sad musical excerpts. Anhedonic depression mediated the relationship between attentional networks recruitment and emotional blunting, and the elevated recruitment of attentional networks during emotional pieces of music carried over into subsequent neutral music. Future studies are needed to investigate whether these findings could be generalised to a clinical population (i.e., major depressive disorder).

## Introduction

Characterized by diminished pleasure or interest and significant impairments in daily functioning and quality of life, depressive disorders are among the most common affective disorders and are considered the largest contributors to global disability^1^. Depressive symptoms are common and vary significantly in severity both in clinical^2^ and non-clinical populations^3^. In fact, in the general population, individual variation in the way and degree to which individuals react to positive and negative emotional stimuli around them has been strongly associated with differences in mental illness and suicidal ideation later in life^4^. In particular, anhedonia, a core symptom of depressive disorders characterized by an inability to experience the pleasure typically associated with pleasant stimuli and life events, has been shown to account for much greater variance in emotional reactivity than low mood^5^. However, to date, the nature of the observed effects (i.e., increased or reduced reactivity to positive and negative cues) remains unclear, especially regarding negative emotional reactivity^5^.

At present, there are three main perspectives on emotional reactivity in depression: (i) attenuated response to positively valenced stimuli (i.e., Positive Attenuation theory), (ii) potentiated reactivity to negatively valenced cues (i.e., Negative Potentiation theory), or (iii) reduced reactivity to both positive and negative information (i.e., Emotion Context Insensitivity; ECI)^6^. In the context of music listening, Vuoskoski and Eerola^7^ found that neuroticism, anger, depression and tension scores correlated positively with perceived sadness in neurotypical adults, supporting Negative Potentiation theory. Similarly, other music studies also observed a positive correlation between neuroticism and sadness, anger and anxiety ratings^8,9^. In contrast, a blunted response to sad music was found in children with greater levels of depressive symptoms drawn from the general population^10^. In a large meta-analysis covering studies which used a range of emotional stimuli such as images, reward tasks and stress tasks, Bylsma et al.^11^ also found evidence for a blunted emotional response to both positive and negative stimuli in Major Depressive Disorder (MDD), providing clear support for the ECI theory^6^. In particular, anhedonia has also been found to significantly predict blunted emotional reactivity to both positive and negative images^5^.

It has been argued that these discrepancies across studies could be explained by the fact that most emotion studies, until recently, have used methods where participants are required to provide a single subjective rating for a given emotional cue^12^. This method has been questioned, as it does not allow for the study of emotional states as they unfold over time^12^. We argue that gaining a better understanding of the interindividual variation in the time-varying behavioural and neural correlates of emotional reactivity in the broader population may, ultimately, lead to identifying markers of vulnerability to affective disorders that could inform early prevention and intervention strategies^13^.

In fact, a growing number of studies have shown that psychological well-being is reflected in the variability of emotional states over time, not just in average emotional states (see^14^ for a meta-analysis). Indeed, the rapidly changing nature of emotions allows human beings to flexibly adapt to their environment by alerting them of important changes around them^14^. As such, exploring how emotions change across time elucidates a process of psychological adjustment and therefore represents a unique window on emotional functioning^14^. Two dynamic measures, the within-subject standard deviation (STD) and the root mean square of successive differences (RMSSD), have been developed to summarize emotional variability^14–18^. While a full description of these metrics can be found in the Methods section, increased STD and RMSSD (i.e., greater intensity and more frequent changes in emotional states) have typically been associated with lower psychological wellbeing (see^14^ for a meta-analysis). However, it is worth noting that these studies employed experience sampling or diary methods (ESM), whereby participants were invited to rate how they felt every few hours throughout the day in the absence of any standardized stimulus, which makes it difficult to make formal comparisons across individuals in terms of their emotional reactivity to specific valence-loaded cues.

In the last decade, neuroimaging studies have provided considerable insight into the neural basis of emotional reactivity as a function of emotional wellbeing in the context of music listening. Using a region of interest analysis, Park et al.^19^ found positive correlations between neuroticism severity and activation in the orbitofrontal cortex, the basal ganglia and the insula in response to happy music. However, their sample size was very small (i.e., 12 participants), and the pieces of music used as a reference had previously been validated to convey a pleasant rather than neutral state, potentially triggering some affective responses. Additionally, the participants were not required to rate how each piece of music made them feel. Instead, they were simply asked to passively listen to the music, making it difficult to establish whether the emotional induction worked. Trait anhedonia has also been shown to negatively correlate with pleasantness ratings and with activity in the nucleus accumbens, the basal forebrain, the hypothalamus, the anterior insula and the orbitofrontal cortex in a music listening task in healthy adults^20^. However, as in most music studies, the subjective ratings here were collected following the scan, using a single rating for each piece of music, which again precludes the examination of the dynamics of emotional reactivity.

In this study of healthy adults, we used the temporal characteristics of music to explore the dynamic features of continuous subjective emotional experience in response to happy, neutral and sad emotional pieces of music as a function of anhedonic depressive symptoms severity. We uniquely combined continuous reporting measures with a novel fMRI analysis method called Leading Eigenvector Dynamics Analysis (LEiDA;^21^). LEiDA is a technique that characterises the dynamics behaviour of brain networks and helps understand changes in brain functional connectivity during the course of a scan. It identifies patterns of neural activity, called states, or clusters, that the brain transitions between over time. The primary outputs for each state are (i) the probability of occurrence (i.e., the percentage of timepoints during which the state dominates during the scan), (ii) the lifetime (i.e., the mean number of consecutive timepoints during which the cluster dominates), (iii) the switching probability (i.e., the probability of switching from one state to another).

LEiDA affords the exploration of dynamic neural processes at the group level while preserving idiosyncratic information about moment-to-moment BOLD fluctuations. Using this approach, greater subclinical depression scores have previously been associated with increased activity of the Default Mode Network (DMN) and fronto-parietal networks, whereas visual and Dorsal Attention networks were diminished in their prominence^22^. However, those findings were observed using resting-state fMRI. It is worth noting that resting-state fMRI is easy to collect and has shed light on different patterns of network dysfunction in specific symptoms of depression, such as atypical frontostriatal and orbitofrontal connectivity in anhedonia^23^. However, recent studies have shown that it is outperformed by naturalistic paradigms when it comes to predicting behaviour^24,25^. Indeed, naturalistic paradigms provide more ecologically valid stimuli that better mimic real-world experiences and engage multiple cognitive and perceptual processes simultaneously, leading to better insights into brain-behaviour relationships compared to the static and less engaging nature of resting-state scans^24^. Resting-state has also been linked to lower test–retest reliability of functional connectivity metrics^26,27^, as well as a difficulty inferring specific mental processes from unconstrained brain activity^28,29^, hence why we decided to focus on rich naturalistic paradigms such as music in this study.

Based on the findings observed in previous emotion and LEiDA studies described above, we hypothesized that we would observe:

- An atypical pattern of emotion dynamics in participants with higher levels of anhedonic depressive symptoms, as evidenced by a significant correlation between their anhedonic depressive scores and the emotional variability metrics (i.e., STD and RMSSD).
- An atypical behaviour of brain networks (i.e., the DMN, fronto-parietal, visual and Dorsal Attention networks) in participants with higher anhedonic depressive symptoms, as evidenced by a significant correlation between anhedonic depressive scores and brain activity metrics (i.e., LEiDA’s probability of occurrence and mean lifetime) for each network.
- An atypical behaviour of these same brain networks (i.e., DMN, fronto-parietal, visual and Dorsal Attention networks) in participants with an atypical pattern of emotion dynamics, as evidenced by a significant correlation between emotional variability metrics (i.e., STD and RMSSD) and brain activity metrics (i.e., LEiDA’s probability of occurrence and mean lifetime) for each network.

Additionally, given the anhedonia-specific atypical brain behaviours observed in previous studies, and the strong association between anhedonia and emotional reactivity, we propose that anhedonia may act as a mediator in the relationship between brain network behaviours and emotional variability. We hypothesized that:

- Atypical behaviours of brain network dynamics (i.e., DMN, fronto-parietal, visual, and Dorsal Attention networks) are associated with anhedonic depressive symptoms.
- Anhedonic depressive symptoms determine emotional variability (i.e., STD and RMSSD).
- Anhedonia mediates the relationship between brain network dynamics and emotional variability, suggesting that brain network dysfunctions lead to altered emotional dynamics via their link to anhedonic symptoms.

## Methods

### Participants

Thirty-nine neurotypical right-handed adults initially participated in the study after providing written informed consent. All participants were recruited based on the following inclusion criteria: between 18 and 30 years of age, good physical health, absence of any psychiatric or neurological disorder, and absence of any MRI counter-indications (i.e., pacemaker, metal in the body, claustrophobia). This age range is commonly used in music studies focussing on emotion processing^7–9^. Furthermore, young adults below 30 years old have been shown to consider music as more important in their lives than adults above 30^30^.

Of the 39 participants who took part, one had incomplete fMRI data and two reported feeling excessively distracted by the background noise generated by the scanner, resulting in an inability to feel emotions that matched those evoked by the songs; these participants were therefore discarded from the analyses. A further three participants had excessive head motion (> 3.3 mm), and another two had more than 15% invalid scans as determined by the CONN toolbox Version 20b (Functional Connectivity toolbox; 31). These participants were also excluded. Consequently, the final number of participants included in the analyses was 31 (17 males and 14 females) (M = 22.6 ± 4.0 years). The study was approved by the King’s College London Human Research Ethics Committee (number HR-19/20-18771) and was carried out in accordance with the Declaration of Helsinki. After data acquisition was completed, the researchers visually inspected the scans to check for artifacts, and a qualified neuroradiologist also reviewed the images to rule out any major neural anomaly, in line with the policies of King’s College London’s Department of Neuroimaging.

### MRI data acquisition

Participants were all scanned in the same 3-Tesla MR scanner (Discovery MR750, General Electric, Milwaukee, WI, USA) at the Centre for Neuroimaging Sciences (Institute of Psychiatry, Psychology and Neuroscience; King’s College London). The MRI data was collected by experienced radiographers using a 12-channel head coil. Anatomical T1-weighted images (MPRAGE) had the following parameters: TR = 7.35 ms, TE = 3.04 ms, flip angle = 11°, slice thickness = 1.2 mm, in-plane resolution 1.05 mm. The functional images were collected using a 2D multi-slice, gradient-recalled Echo-Planar Imaging (EPI) sequence with the following parameters: TR = 2 s, TE = 33 ms, flip angle = 75°, slice thickness = 3 mm, field of view = 240 mm, 64 × 64 matrix. EPI is an advanced MRI technique that enables fast acquisition of multiple images of the brain, using rapid variations in the magnetic field gradients to capture detailed images of the brain in a matter of seconds. The total duration of the fMRI run was 8 min and 27 s. Four dummy scans were acquired at the beginning of the time series, to allow the signal to achieve steady state. Those four volumes (i.e., the first 8 s) were not part of the analysed time series. All participants were provided with earplugs and padded headphones to limit any discomfort deriving from the background noise generated by the scanner.

### MRI data pre-processing

The data were pre-processed using the CONN toolbox Version 20b^31^, MATLAB R2020a (MathWorks, Natick, MA, USA). The pre-processing consisted of realignment to correct for volume-to-volume head motion, co-registration of the functional data to the anatomical image, and spatial normalization into the Montreal Neurological Institute (MNI) standardized space using the parameters generated during segmentation of the T1 weighted structural image. Finally, the normalized functional MRI data were smoothed with full width at half-maximum isotropic Gaussian kernel of 8 mm. The artifact rejection tool (ART), implemented in CONN (http://www.nitrc.org/projects/artifact_detect), was used to detect outlier volumes in the timeseries with respect to head motion and global signal changes. One covariate for each outlier volume was then entered in the denoising step to lessen the contribution of those scans to the results of the fMRI analyses. Finally, white matter and CSF signal were extracted and broken into components, capturing major sources of variance, using principle component analysis, and the resulting components were regressed out of the data. This anatomical component-based noise correction approach (aCompCor;^32^) was also used to ensure that physiological and other sources of noise which were unlikely to be neuronal in origin did not confound the subsequent connectivity analysis.

### Stimuli and procedures

During the fMRI acquisition, all participants were required to listen to 13 pieces of classical music and asked to continuously rate how each piece of music made them feel on a scale of − 6 (very sad) to + 6 (very happy). These pieces of music were chosen as they have previously been identified as eliciting happy, neutral and sad emotional states in healthy adults^33^. The participants listened to the auditory stimuli through headphones, and they responded by using a two-button box. Pressing the left button made the slider move left towards − 6, while pressing the right button made it move right towards + 6. The equipment was tested before the scan started to ensure auditory clarity. All songs were presented in the exact same order for all participants to facilitate analysis methods, with 2 s of silence separating each pair of pieces of music. Overall, the run consisted of 3 happy songs, 3 sad songs and 7 neutral songs. It started with a neutral song, and then each sad song and each happy song was followed by a neutral song. The name of each musical piece, the name of the composer, the order of presentation, the song type and the start and end times are presented in Table 1. The pieces of music slightly varied in length to minimise abrupt and harsh stops.

**Table 1:** Names of each music piece, order of presentation, start and end times, composer and song types.

Order of presentation	Start time (s)	End time (s)	Musical pieces	Composer	Song type
1	0	29	Water music—passepied	Handel	Neutral
2	31	65	Kol Nidrei	Bruch	Sad
3	67	109	Violin romance no.2 in F major	Beethoven	Neutral
4	111	147	Radetzky march	Strauss	Happy
5	149	183	L’oiseau prophete	Schumann	Neutral
6	185	225	Suite for violin and orchestra in A minor	Sinding	Sad
7	227	259	Clair de lune	Debussy	Neutral
8	261	301	A little night music—allegro	Mozart	Happy
9	303	337	Water music menuet	Handel	Neutral
10	339	381	Concerto de Aranjuez—Adagio	Rodrigo	Sad
11	383	425	The Planets—Venus	Holst	Neutral
12	427	467	A little night music—Rondo Allegro	Mozart	Happy
13	469	507	La Traviata—Prelude to the 1st scene	Verdi	Neutral

#### Self-report measures

Prior to the scan, participants were required to fill in questionnaires aimed at assessing their familiarity with each piece of music, their music background, anhedonic depression severity, neuroticism, rumination levels and trait emotional reactivity (see details below).

##### Personality and mental health questionnaires

The participants were then required to fill in the following questionnaires: the anhedonic depression subscale of the Mood and Anxiety Symptoms Questionnaire (MASQ-AD;^34^), the neuroticism subscale of the Big Five Inventory (BFI;^35^),the rumination subscale of the Rumination-Reflection Questionnaire (RRQ;^36^) and the short version of the Perth Emotional Reactivity Scale (PERS-short; (^37^).

#### Mood and anxiety symptoms questionnaire-anhedonic depression subscale (MASQ-AD)

The 14-item MASQ-AD subscale of the MASQ was chosen to evaluate participants’ levels of anhedonic depression^34^. This subscale has previously been used to examine the neural basis of anhedonic depression in the context of music listening, and it was specifically chosen for this study because it has demonstrated satisfactory variance and high sensitivity to individual differences in a healthy adult population^20^. It comprises items that assess loss of interest and enjoyment concerning a wide-range of everyday situations. For each item, participants were required to indicate on a five-point scale (1 = not at all, 5 = extremely) to what extent each statement applied to them. Scores were then summed up across all 14 items, and the overall scores ranged between 14 and 70. A score of 14 indicates an absence of anhedonic symptoms, while a score of 70 reflects high levels of anhedonic depression. The MASQ-AD has shown good internal consistency alpha in university samples (α = 0.80;^38^).

For this paper, we focussed on anhedonic depressive symptoms and therefore only the MASQ-AD and key associated findings are fully described in this paper. More information about the other scales can be found in Supplementary Material 1a.

##### Familiarity ratings

The participants were first required to listen to each piece of music through the Lime Survey online tool (limesurvey.org) and rate them in terms of how familiar they were with each of them on a five-point scale (1 = I have never heard this piece of music before, 5 = I know this piece of music very well). Lime Survey is an open-source platform that allows users to design customizable surveys and collect data freely online. The mean and standard deviation of the familiarity ratings were then calculated for each song across all participants.

##### Music background

The music background questionnaire consisted in four statements which the participants were asked to rate in terms of how much they each applied to them (1 = not at all, 5 = extremely): “I enjoy listening to music” (question 1), “I enjoy listening to classical music” (question 2), “I have a broad knowledge of classical music (e.g., I can recognise and name different classical music pieces, composers, instruments etc.)” (question 3), and “I can play a musical instrument” (question 4).

### Behavioural analyses

For all behavioural analyses, normality of each variable (e.g., MASQ-AD, STD, RMSSD, familiarity ratings and music background questions) was tested using the Shapiro–Wilk test (significance was set at 0.05).

#### Familiarity ratings and music background

Pearson’s correlations were carried out between music background questions and summary measures of the familiarity ratings for each song type (i.e., Familiarity_H for the familiarity with the happy songs, Familiarity_S for the familiarity with the sad songs, Familiarity_N for the familiarity with the neutral songs). Question 3 (i.e., I have a broad knowledge of classical music) consistently exhibited strongest associations with all four familiarity summary measures, therefore the question 3 scores were added to gender and age as a nuisance covariate for the subsequent analyses. Full details are provided in Supplementary Material 2a.

From this point, reference to music background/familiarity will refer to the responses to question 3.

#### MASQ-AD and mean subjective ratings

We carried out a repeated-measures ANOVA, followed by two post-hoc paired t-tests to explore whether there were significant differences between mean subjective ratings for ‘happy’, ‘neutral’ and ‘sad’ pieces of music. We then correlated mean ratings with MASQ-AD scores to explore associations between the anhedonic depression symptoms and mean subjective ratings for each song type. Full details are provided in Supplementary Material 3a.

In all cases, the threshold for significance was set at p < 0.05.

#### MASQ-AD and emotional blunting/variability

For each participant, we calculated two metrics representing emotional blunting/variability: the within-subject standard deviation (STD) and the within-subject root mean square of the successive differences (RMSSD). The STD provides information about the variance of the participant’s emotional states across the entire task, where high and low values reflect heightened emotional reactivity and blunted responses, respectively^17^. The RMSSD reflects moment-to-moment fluctuations in emotional reactivity across the entire task, where higher scores reflect greater emotional lability, characterized by more significant emotional swings from one moment to the next, while lower scores suggest a less flexible emotional response^18^. For each participant, the STD and the RMSD were calculated as follows, in line with Jahng, Trull and Wood^39^, where n is the number of timepoints, $\upmu$ is the average of the subjective ratings over the entire task, x_i_ is the subjective rating at time t, and x_i+1_ is the subjective rating at time t + 1:

$$\text{STD }=\sqrt{\frac{\sum {\left({x}_{i}-\upmu \right)}^{2}}{n}}$$

$$\text{RMSSD }= \sqrt{\frac{\sum_{i=1}^{n-1}{\left({x}_{i+1}-{x}_{i}\right)}^{2} }{n-1}}$$

To identify whether anhedonic depression was related to either of the participants’ emotional blunting/variability metrics, we then calculated Pearson’s correlation coefficients between the MASQ-AD and the within-subject STD and RMSSD metrics, controlling for age, gender and music background/familiarity.

### LEiDA analyses

The LEiDA analyses were carried out in MATLAB R2020a (MathWorks, Natick, MA, USA) using scripts adapted from Cabral et al., 2017 (^21^; https://github.com/juanitacabral/LEiDA). N = 105 regions of interest (ROI) defined anatomically based on the Harvard–Oxford cortical atlas were extracted from the CONN toolbox^31^. In line with previous work by Cabral et al.^22^, the cerebellar ROIs were excluded from the analyses because of the absence of cerebellar networks within the Yeo parcellation used for this study^40^. The Yeo parcellation is widely used in neuroimaging research and typically divides the brain into seven meaningful functional networks, including the default-mode network, the fronto-parietal network, the limbic network, the visual network, the somato-motor network, the ventral attention network and the dorsal attention network^40^.

**Figure 1:**
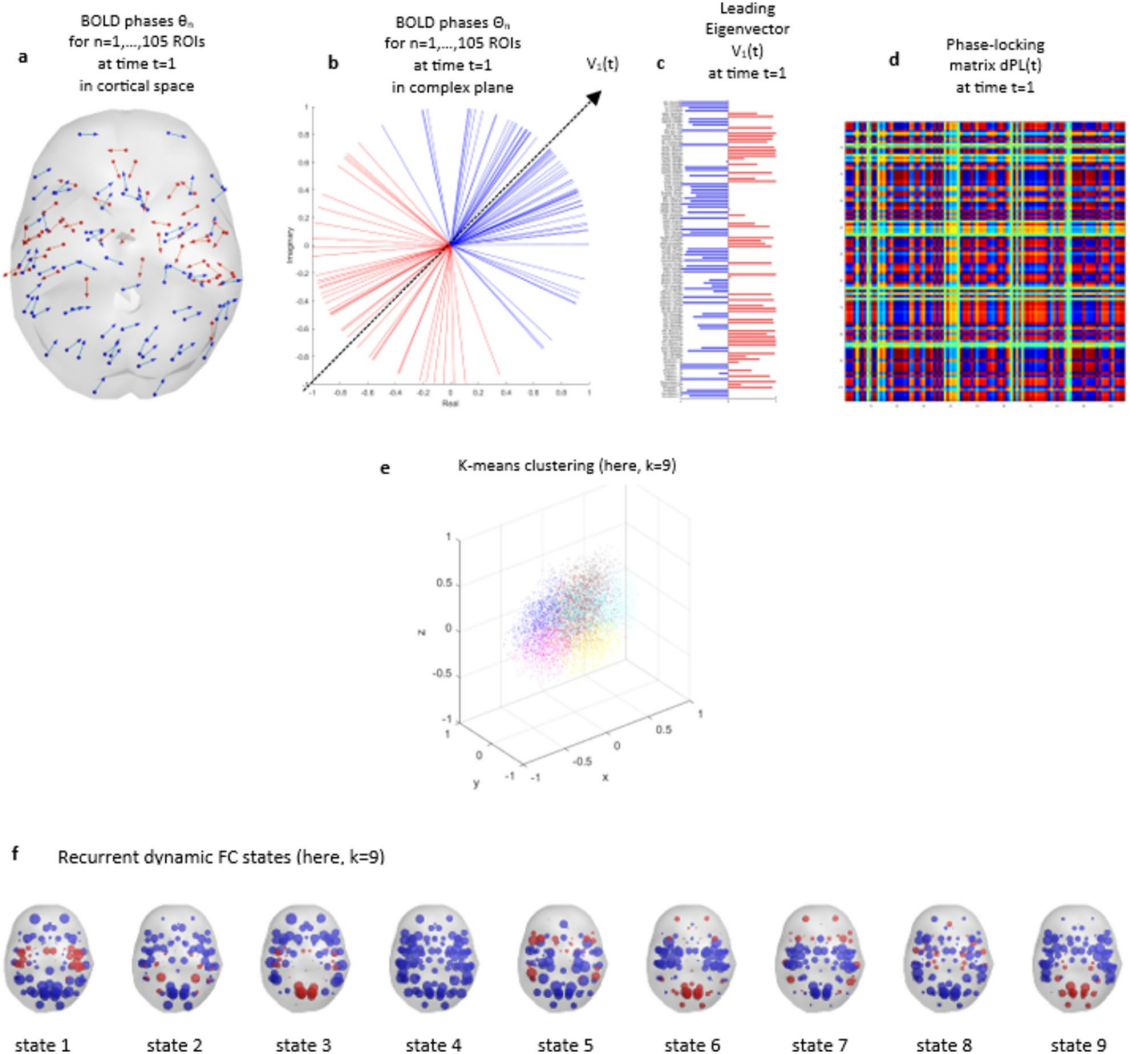
Identification of recurrent phase-locking patterns (or states) in fMRI signals. At each timepoint (here, first volume t = 1), Bold phases of each ROI are represented in (**a**) cortical space, where each arrow reflects the phase orientation of a given ROI and is originated from the centre of gravity of that ROI, and (**b**) in complex plane, where each arrow is centred at the same origin and the leading eigenvector V_1_ is represented as a dashed arrow. Phases are divided into two communities (i.e., blue or red) depending on the direction they project onto V_1_. (**c**) Each element in the horizontal bar plot captures the relative contribution of a given ROI to V_1_ at a given timepoint (here, first volume t = 1). (**d**) The 105 × 105 dynamic phase-locking matrix dPL(t) reflects the degree of alignment, or synchrony, between pairs of ROI phases at time t (here, first volume t = 1). The warmer the colour, the more synchronised the ROIs. For each participant, we obtain a leading eigenvector V_1_ at each timepoint t. (**e**) All leading eigenvectors V_1_ across all timepoints and all participants are then divided into k clusters using k-means clustering (here, k = 9). (**f**) Each cluster, or state, is illustrated in cortical space. Each sphere reflects the centre of gravity of a given ROI and is coloured depending on the direction it projects onto the leading eigenvector of that state.

Full details of the implementation of the LEiDA method can be found in Cabral et al.^21^, but in brief: for each ROI, the timeseries of the blood oxygen level dependent (BOLD) signal was first Hilbert-transformed to create an analytic signal which captures the time-varying phase of the BOLD oscillations. We then calculated the degree to which BOLD phases were synchronised between pairs of ROIs at each timepoint t, as reflected in the dynamic phase-locking matrix dPL(t) (Fig. 1).

The next step consisted in calculating a leading eigenvector V_1_(t) for each dynamic phase-locking matrix dPL(t) to identify recurrent patterns in the dPL with reduced dimensionality. The leading eigenvector V_1_(t) is the vector associated with the largest eigenvalue of the dPL(t) matrix, capturing the most significant pattern of variance in the data. It typically highlights the dominant, or strongest, pattern of synchronization between different regions of the brain at a given timepoint (t). Focussing on this primary pattern reduces the complexity of the data, while retaining the most important information about brain activity dynamics. In particular, V_1_(t) contains N elements (i.e., brain regions) which each have either a positive or a negative sign depending on the direction their phase projects onto V_1_(t). When all elements of V_1_(t) have the same sign (i.e., negative), then all phases are pointing in the same direction and are considered globally synchronised, which reflects global coherence mode. In contrast, a positive sign reflects meaningful functional networks whose phases detach from the global coherence mode and dominate at a given timepoint^41,42^. These phases are in synchrony between themselves and out of synchrony with the rest of the brain^41,42^.

We then used k-means clustering to group similar brain activity patterns into distinct clusters, or states, based on their characteristics. K-means clustering iteratively clusters all leading eigenvectors V_1_(t) into k = 5 to k = 10 network states. In essence, the method attempts to identify the optimal (k) number of states such that the sum of squared differences between the data points and the k centroids is minimised (i.e., the centroids are placed such that the data point clusters are maximally separated). The Dunn score was then calculated for each clustering attempt, each with a different k-value, to identify the number of states k that best explained the data.

Once the optimal number of states k was identified, all three LEiDA metrics were calculated for each cluster or state (i.e., the probability of occurrence, the lifetime and the switching probability).

To assign to each state a meaningful reference label based on known functional networks, we then calculated the spatial similarities between each state and seven resting-state networks previously identified by Yeo et al.^40^. This consisted in computing the Pearson’s correlation coefficients between each of the seven networks and the centroids V_k_ previously obtained from the k-means clustering analysis, following the methodology described in Vohryzek et al.^42^. Significance was set at p < 0.01/k.

For all LEiDA analyses, correction for multiple comparisons was implemented for each metric using False Discovery Rate (FDR) correction^43^, and age, gender and music background/familiarity were controlled for. FDR correction attempts to minimise the proportion of positive results likely to be false positives, typically below 5%.

#### Across the entire task

##### MASQ-AD and LEiDA metrics

We first investigated whether the severity of anhedonic depression symptoms was associated with each of the LEiDA measures across the entire task by calculating the Pearson’s correlation coefficients between the MASQ-AD and each LEiDA metric for each identified state.

##### Emotional blunting/variability and LEiDA metrics

We then explored whether each of the LEiDA metrics was associated with emotional blunting/variability by calculating the Pearson’s correlation coefficients between each LEiDA metric and the within-subject STD, and between each LEiDA metric and the within-subject RMSSD for each state.

##### Mediation analysis

To better understand the relationship between the LEiDA metrics, MASQ-AD scores and emotional blunting, we carried out a mediation analysis using IBM SPSS Statistics version 29 and macro-programme PROCESS version 4.2^44^. We used the LEiDA metrics as an independent variable (IV), emotional blunting as a dependent variable (DV) and MASQ-AD as the mediator (M). A series of regression models were then fitted. The first step consisted in predicting M using IV (i.e., a-path) and then predicting DV using M (i.e., b-path). The second step consisted in measuring the indirect effect of IV on DV through M, by multiplying a and b. This allowed us to explore how M mediates the relationship between IV and DV. In order to investigate whether the mediation model was partial or full, we also estimated the direct effect of IV and DV, as well as the total effect.

#### Focussing on each song type

Previous studies have found a stronger brain/behaviour relationship during less emotional moments of a naturalistic task in depressed adolescents^45^, and carry-over effects into subsequent conditions have also been previously observed in the context of sad emotional states^46^ and lethargy^47^ in depression. Therefore, we decided to explore further whether the relationship between the anhedonic depressive symptoms and the probability of occurrence of a given state was related to the valence of the song being played. We first extracted LEiDA metrics for each song type, only for the states where a significant correlation was observed between the MASQ-AD and their LEiDA metrics across the entire song. For each relevant state, each LEiDA metric was averaged across all songs within the same song type. We then calculated the Pearson’s correlation coefficients between the MASQ-AD and each of the LEiDA measures of a given state for each song type.

## Results

### Behavioural results

All of the behavioural variables (e.g., MASQ-AD, STD, RMSSD, familiarity ratings and music background questions) were normally distributed, as reflected by a non-significant Shapiro–Wilk normality test (p > 0.05).

#### Descriptive statistics

##### Personality and mental health questionnaires

The mean and between-subject standard deviation for the Mood and Anxiety Symptoms Questionnaire (MASQ-AD) were 34.26 and 8.843, respectively. Descriptive statistics representing the mean and between-subject standard deviation for the other personality and mental health questionnaires can be found in Supplementary Material 1b, alongside correlations between each pair of questionnaires, and associated p-values.

We chose to focus on the MASQ-AD subscale for the rest of the analyses because the focus of this study was on anhedonic depressive (AD) symptoms, and because of the highly correlated scores between MASQ-AD and the other four questionnaires.

##### Subjective ratings and familiarity ratings

Descriptive statistics representing the mean and between-subject standard deviation of the subjective ratings for each song can be found in Table 2. Subjective ratings ranged between − 6 (very sad) to + 6 (very happy), and familiarity ratings ranged between 1 (I have never heard this piece of music before) and 5 (I know this piece of music very well).

**Table 2:** Descriptive statistics providing the mean and between-subject standard deviation (SD) of the subjective ratings and familiarity ratings for each song.

Musical pieces	Mean (SD) subjective ratings	Mean (SD) familiarity ratings
Happy pieces		
Radetzky march	3.92 (1.55)	2.65 (1.43)
A little night music—allegro	3.75 (1.67)	3.10 (1.47)
A little night music—Rondo Allegro	3.24 (1.60)	2.13 (1.12)
Sad pieces		
Kol Nidrei	− 2.06 (2.06)	1.48 (0.89)
Suite for violin and orchestra in A minor	− 2.98 (1.59)	1.39 (0.72)
Concerto de Aranjuez—Adagio	− 2.48 (1.88)	1.77 (1.20)
Neutral pieces		
Water music—passepied	1.28 (2.03)	2.26 (0.96)
Violin romance no.2 in F major	1.15 (2.24)	2.29 (1.16)
L’oiseau prophete	− 0.05 (1.50)	1.29 (0.64)
Clair de lune	− 0.61 (2.83)	3.45 (1.41)
Water music menuet	1.15 (1.38)	1.77 (1.06)
The Planets—Venus	− 1.62 (1.91)	1.74 (1.21)
La Traviata—Prelude to the 1st scene	1.49 (1.72)	2.06 (1.09)

Participants (n = 31) were required to continuously rate each song in terms of how they made them feel, from − 6 (very sad) to + 6 (very happy) and were also asked to indicate how familiar they were with each song (1 = I have never heard this piece of music before, 5 = I know this piece of music very well).

#### Familiarity ratings and music background

Full details about the mean and standard deviation of the music background questions and familiarity ratings for each song type can be found in Supplementary Material 2b.

Full details about the coefficients of determination and correlation coefficients between the music background questions and the familiarity scores are also provided in Supplementary Material 2b.

As question 3 consistently exhibited the highest coefficients of determination and correlation coefficients for the four familiarity summary measures, the question 3 scores were used as a nuisance covariate for the subsequent analyses.

#### MASQ-AD and mean subjective ratings

Mean ratings were significantly higher for happy compared to neutral songs and significantly lower for sad compared to neutral songs. Additionally, MASQ-AD significantly negatively correlated with happy ratings, and significantly positively correlated with sad ratings. Significance was set at 0.05. Full details are provided in Supplementary Material 3b.

#### MASQ-AD and emotional blunting/variability

**Figure 2:**
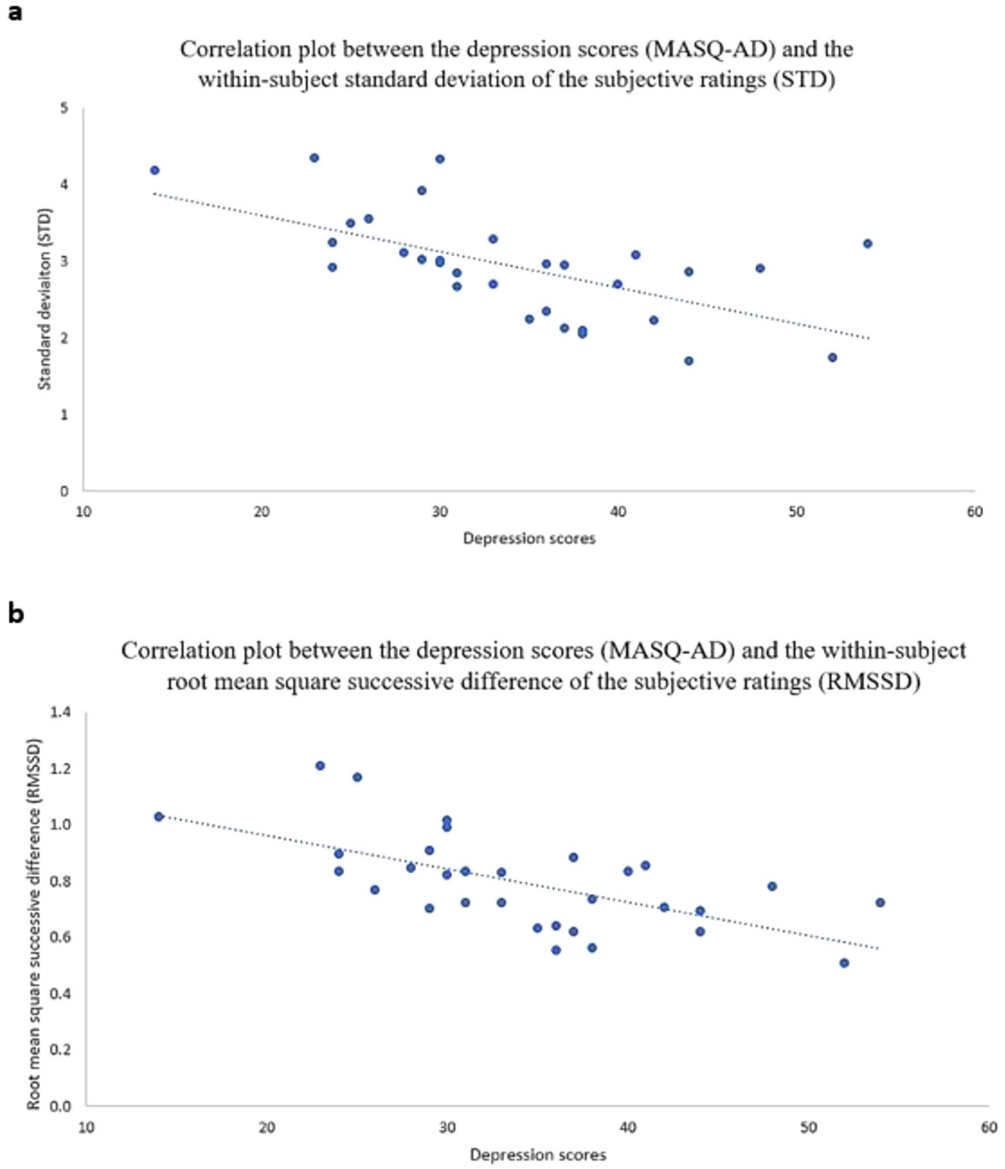
Scatter plots representing the depression scores (MASQ-AD; x axis) and (**a**) the within-subject standard deviation of the subjective ratings (STD; y axis) and (**b**) the within-subject root mean square successive difference of the subjective ratings (RMSSD; y axis), with trendlines.

There was a large, negative, correlation between the MASQ-AD and the within-subject STD of the subjective ratings (r = − 0.630, pFDR < 0.001), and between the MASQ-AD and the within-subject RMSSD of the subjective ratings (r = − 0.656, pFDR < 0.001). An illustration is provided in Fig. 2a,b.

It is worth noting that pFDR represents the p-value after False Discovery Rate (FDR) correction^43^.

**Figure 3:**
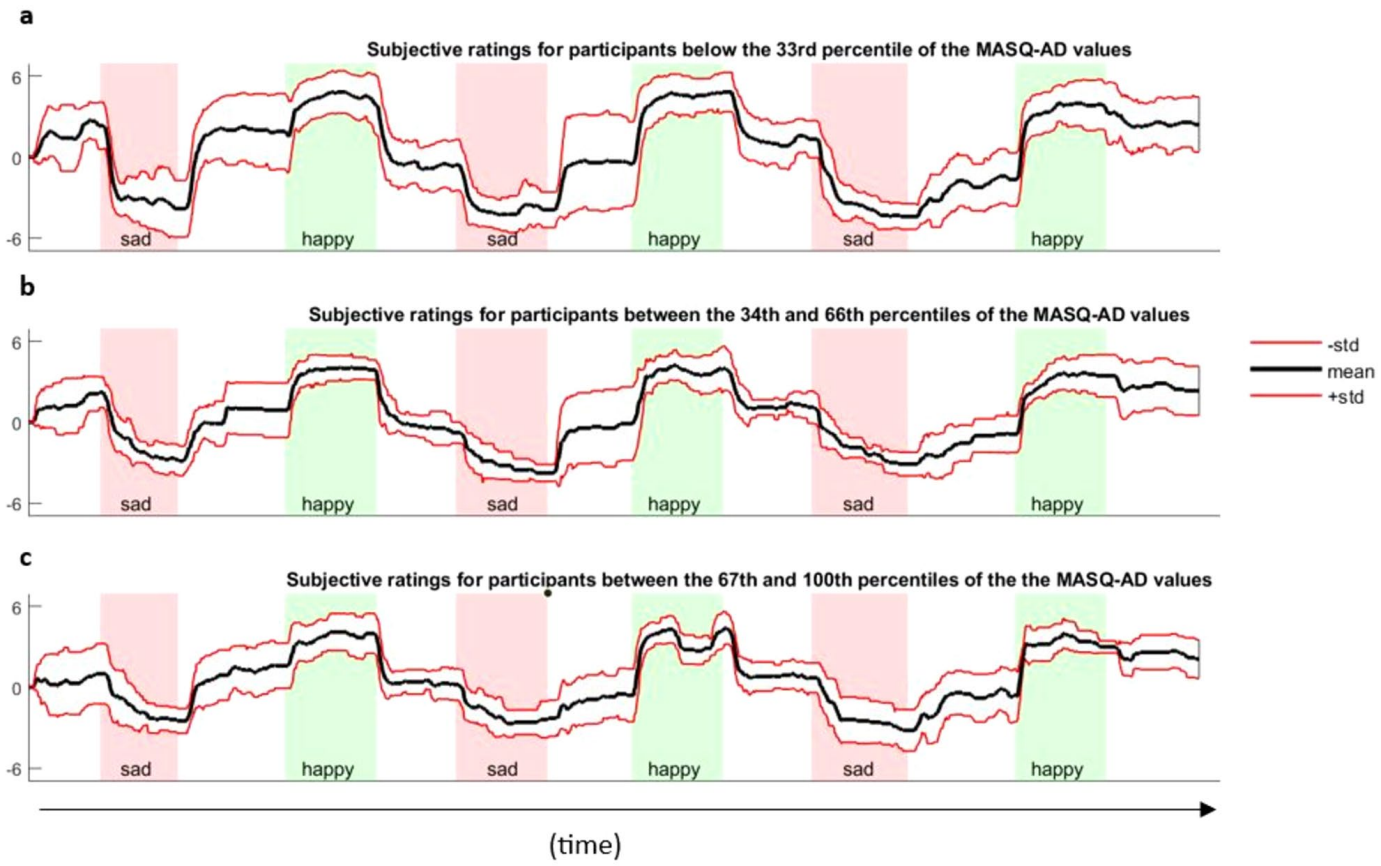
Continuous subjective ratings throughout the entire music task, averaged across the participants with an MASQ-AD score (**a**) in the bottom 33% of the MASQ-AD values; (**b**) between the 34th and the 66th percentiles of the MASD-AD values; and (**c**) in the top 33% of the MASQ-AD values. The bold lines refer to the averaged subjective ratings. The red lines represent the standard deviation associated with the mean subjective ratings.

Figure 3 illustrates the lower amplitude (i.e., lower STD) in subjective ratings displayed by participants whose MASQ-AD scores fell into the top 33% of the range of MASQ-AD values (i.e., (c) higher levels of anhedonic depressive symptoms) compared to those in the middle 33% (i.e., (b)), and those in the bottom 33% (i.e., (a) lower levels of anhedonic depressive symptoms). Participants higher in anhedonic depressive symptoms (i.e., bottom panel c) displayed blunted, or less intense, responses to emotional pieces of music, as evidenced by higher ratings for sad songs and lower ratings for happy songs. This translated into lower within-subject emotional variability. In contrast, participants lower in anhedonic depressive symptoms (i.e., top panel a) displayed higher emotional variability and experienced more intense emotional responses, as evidenced by more pronounced emotional highs and lows.

### LEiDA results

**Figure 4:**
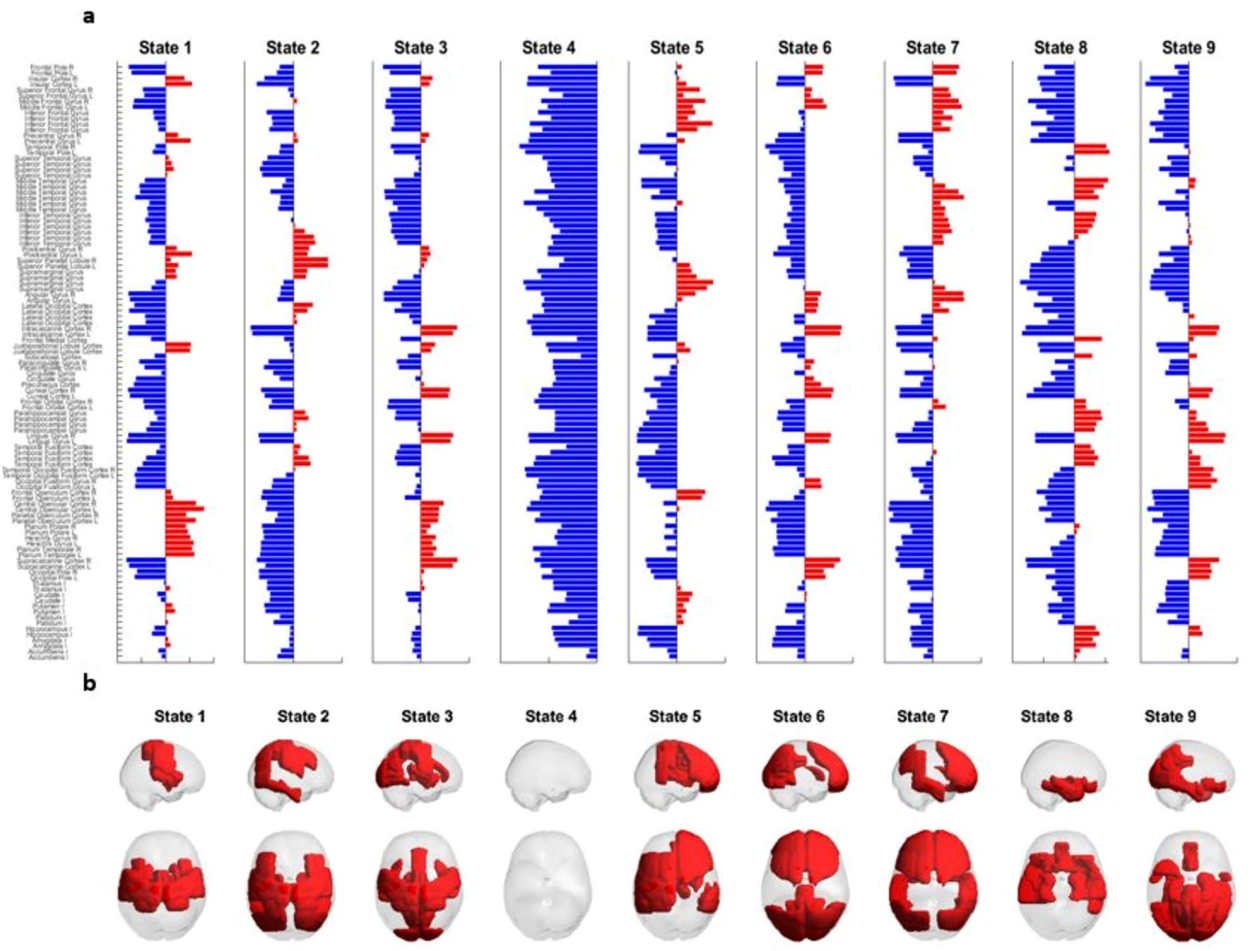
Repertoire of the states deriving from the optimal clustering solution k = 9. Top panel (**a**) refers to the contribution of each brain region to each state. A blue colour represents a negative projection, while a red colour refers to a positive projection. Bottom panel (**b**) represents the rendering of all brain regions with positive projections onto the leading vector of that state.

K-means clustering analyses revealed an optimal solution of 9 brain states based on the respective Dunn scores for the range of k-values we used. Figure 4 illustrates the direction and degree to which each of the 105 ROIs’ phases projects onto the leading vector V_1_ of each state (a), and the rendering of each of these states on the cortex (b). In the top panel (a), the brain regions highlighted in red represent the brain regions that positively project onto V_1_, for a given state. These same brain regions are then rendered in red on the cortex in the bottom panel (b).

#### Correlations between each LEiDA state and each Yeo network

State 4, which occurred 14% of the time on average, did not significantly correlate with any Yeo network and can therefore be considered as the global coherence network where all the ROIs’ phases point in the same direction (i.e., negative sign represented by blue bars in Fig. 4a). In contrast, the other eight states are each made up of a specific set of ROIs whose phases have a positive sign (illustrated in red in Fig. 4a,b) and are in synchrony between themselves but out of synchrony with the rest of the ROIs. This limited group of ROIs detaches from the global coherence network and forms a meaningful network that spatially overlapped with one or more Yeo networks^40^. State 1, which occurred 13% of the time, significantly correlated with the somato-motor and ventral attention networks of Yeo et al. (2011) (r = 0.72 and 0.47, respectively); state 2 (9%), with the Dorsal Attention Network (DAN; r = 0.58); state 3 (9%), with the visual and somato-motor networks (r = 0.43 and 0.42, respectively); state 5 (12%), with the ventral attention and fronto-parietal networks (r = 0.47 and 0.53); state 6 (9%), with the visual network (r = 0.50); state 7 (11%), with the fronto-parietal and DMN networks (r = 0.60 and 0.56, respectively); state 8 (11%), with the limbic network (r = 0.60); and state 9 (12%), with the visual network (r = 0.73).

#### LEiDA metrics across the entire task

##### MASQ-AD and LEiDA metrics

**Figure 5:**
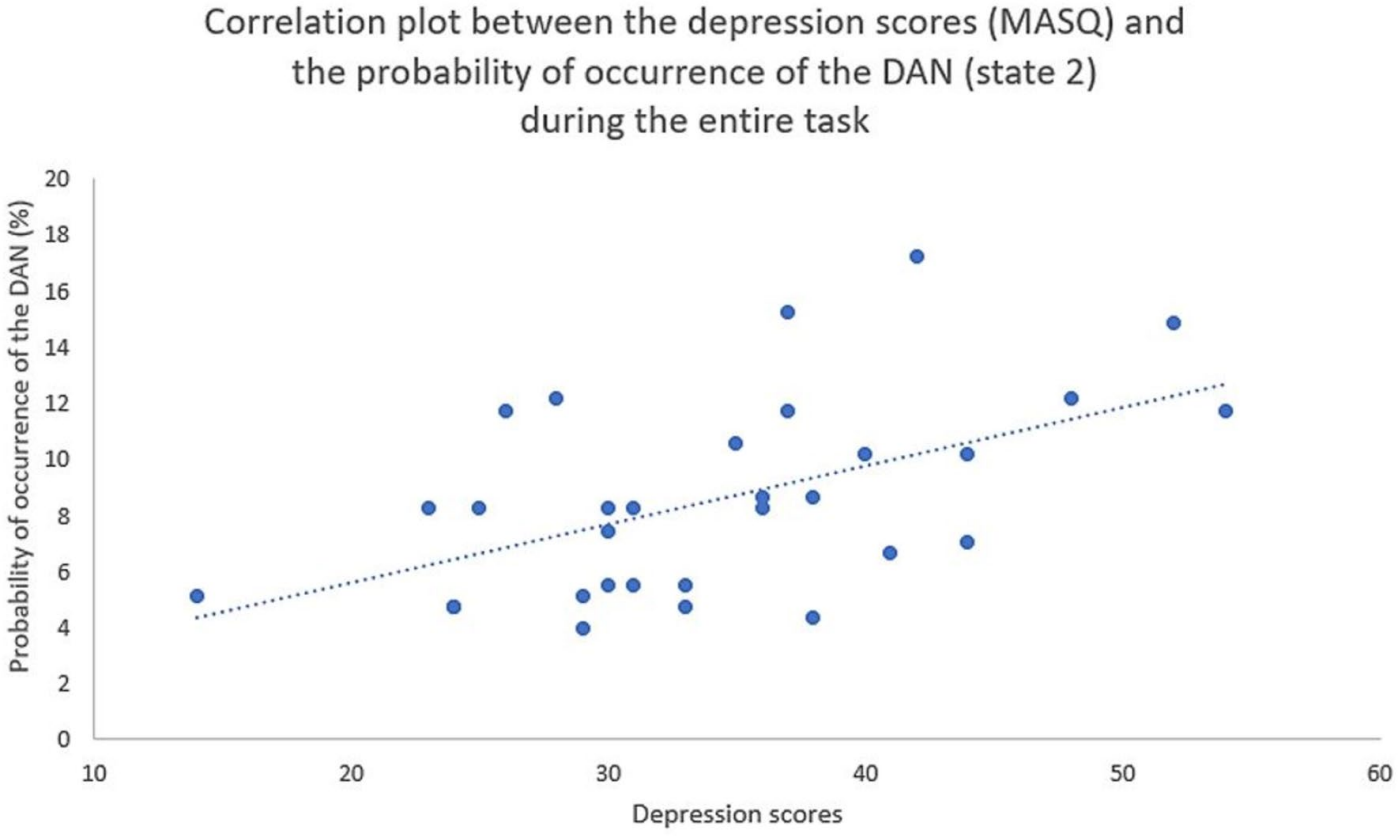
Scatter plot representing the depression scores (MASQ-AD; x axis) and the probability of occurrence of the dorsal attention network (DAN; state 2; y axis), with trendline.

*Probability of occurrence *A significant positive correlation was identified between the MASQ-AD (i.e., anhedonic deression) questionnaire and the probability of occurrence of state 2 (i.e., Dorsal Attention Network; DAN) (r = 0.59, pFDR = 0.009; Fig. 5). There was no significant correlation between MASQ-AD and the probability of occurrence of the other states.

*Lifetime *There was no significant correlation between MASQ-AD and the lifetime of any of the states.

*Switching probability *There was a significant positive correlation between MASQ-AD and the probability of switching from the global coherence network (state 4) to the DAN (state 2) (r = 0.544; p_uncorr_ = 0.003). We also found a significant positive correlation between the MASQ-AD and the probability of switching from the visual network (state 6) to the ventral attention and fronto-parietal networks (state 5) (r = 0.480; p_uncorr_ = 0.011) and from the fronto-parietal and DMN networks (state 7) to the ventral attention and fronto-parietal networks (state 5) (r = 0.384; p_uncorr_ = 0.048). However, none of these correlations survived FDR correction type 1 error control for multiple comparisons (pFDR > 0.05).

##### Emotional blunting/variability and LEiDA metrics

**Figure 6:**
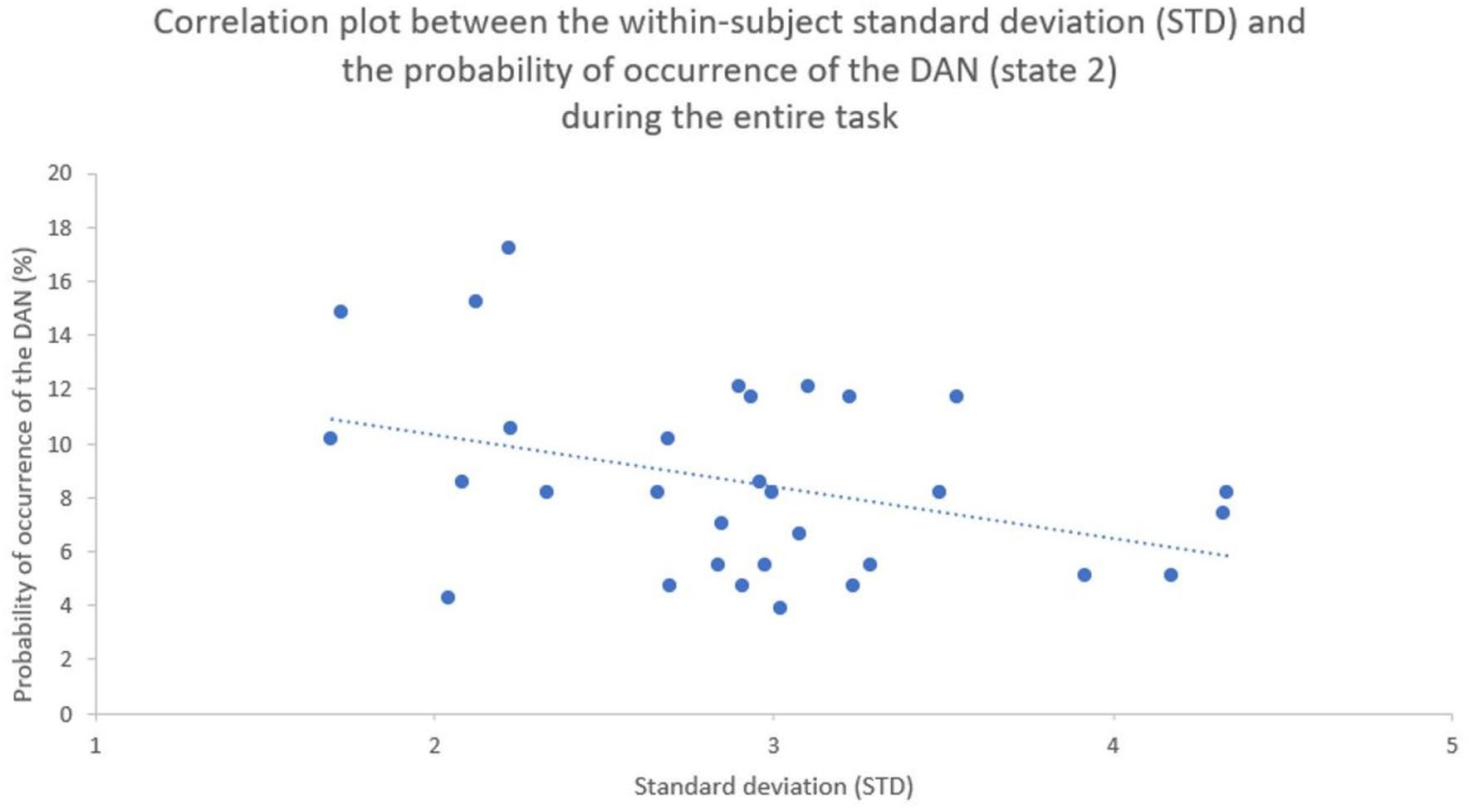
Scatter plot representing the within-subject standard deviation (STD; x axis) and the probability of occurrence of the dorsal attention network (DAN; state 2; y axis), with trendline.

There was a significant negative correlation between the probability of occurrence of the DAN (state 2) and the within-subject STD (i.e., spread in the subjective ratings) (r = − 0.393, pFDR = 0.043), and a non-significant negative correlation between this same network state (i.e., state 2) and the within-subject RMSSD (r = − 0.348, pFDR = 0.076) (Fig. 6).

##### Mediation

The first step of our mediation analysis showed that the probability of occurrence of the DAN significantly positively predicted anhedonic depressive symptoms (i.e., MASQ-AD; a = 0.54, p = 0.0012) which, in turn, significantly negatively predicted the within-subject standard deviation of the subjective ratings (i.e., STD; b = − 0.62, p = 0.0048).

**Figure 7:**
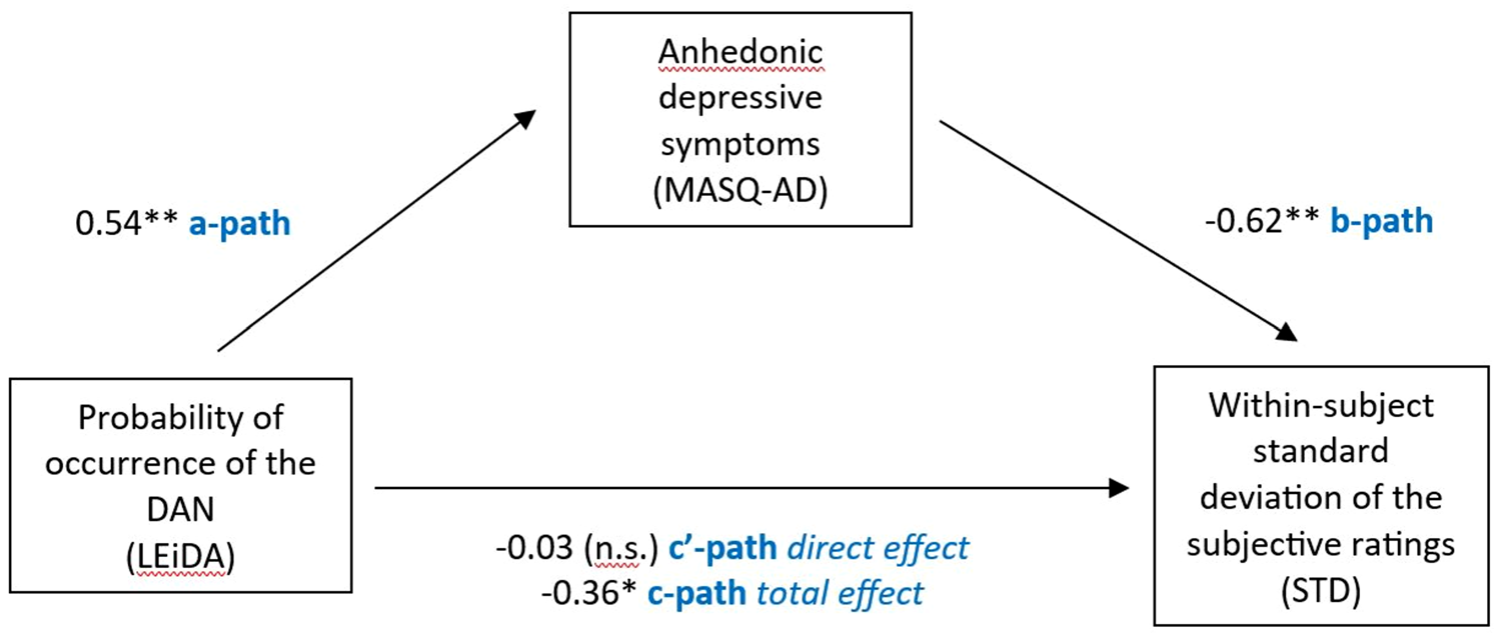
Summary findings of the mediation analysis displaying standardized regression coefficients (betas) and their level of significance for each analysis: *p < 0.05; **p < 0.01; *n.s.* non-significant. We found that anhedonic depressive symptoms fully mediated the relationship between the probability of occurrence of the dorsal attention network (i.e., DAN) and the within-subject standard deviation of the subjective ratings (i.e., STD).

Step 2 revealed a significant indirect effect of the probability of occurrence of the DAN on STD, through MASQ-AD (i.e., ab = − 0.33, bootstrapped confidence interval [− 0.66 − 0.04]). Additionally, the direct effect of the probability of occurrence of the DAN on STD was non-significant (c’ = − 0.03, p > 0.05), while the total effect was significant (c = − 0.36, p = 0.0425). Taken together, these findings demonstrate that anhedonic depressive symptoms fully mediated the relationship between the probability of occurrence of the DAN and STD (Fig. 7).

Here, we used the bootstrap test because it is known as the state-of-the-art method for testing indirect effects in mediation models and providing robust, reliable confidence intervals^48^. In short, bootstrapping in mediation analysis is a commonly used statistical method that estimates the indirect effect of an independent variable on a dependent variable through a mediator, by repeatedly resampling with replacement from the data to obtain accurate confidence intervals for this effect.

#### LEiDA metrics with a focus on specific song types

Additional analyses revealed a significant positive correlation between the MASQ-AD and the probability of occurrence of state 2 (i.e., DAN) during neutral songs overall (r = 0.606, pFDR < 0.001). There was also a significant positive correlation between the MASQ-AD and the probability of occurrence of state 2 (i.e., DAN) during neutral-following-sad songs (r = 0.698, pFDR < 0.001) and a significant positive correlation between the MASQ-AD and the probability of occurrence of state 2 during neutral-following-happy songs (r = 0.459, p_uncorr_ = 0.016), however the latter did not survive FDR correction.

**Figure 8:**
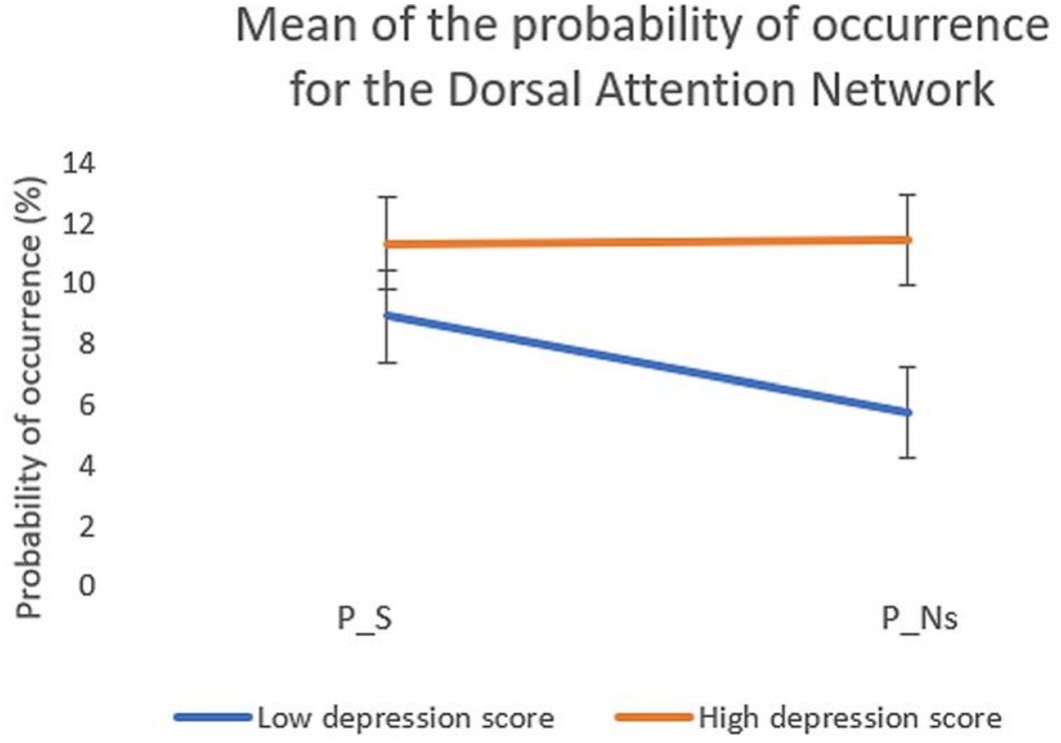
Mean and standard deviation of the probability of occurrence during sad songs (P_S) and during neutral-following-sad songs (P_Ns) for participants low in depression (MASQ-AD score < 34; blue) and high in depression (MASQ-AD score ≥ 34; orange).

To better understand how individuals with higher anhedonic depressive symptoms differ from those with lower anhedonic depressive symptoms in the way they transition from sad songs to neutral songs, we divided our participants into two groups: participants with a score above the median score on MASQ-AD (i.e., 34) and those below the median score. Figure 8 illustrates that the mean of the probability of occurrence of the DAN decreased in the transition from sad songs (P_S) into neutral-following-sad songs (P_Ns) for participants lower in anhedonia scores, while it remained elevated for participants higher in anhedonia scores.

## Discussion

In line with our first hypothesis, we observed an atypical pattern of emotion variability (i.e., STD and RMSSD metrics) in participants with higher levels of anhedonic depressive symptoms (i.e., MASQ-AD). Blunted emotional reactivity, as described by the Emotion Context Insensitivity (ECI;^6^) theory, aligns with our findings showing negative correlations between MASQ-AD and both STD and RMSSD, a negative correlation between MASQ-AD and the ‘happy’ beta (i.e., averaged ratings during happy songs), and a positive correlation between MASQ-AD scores and the ‘sad’ beta (i.e., averaged ratings during sad songs). However, these findings are inconsistent with the ESM studies reviewed by^14^. One could argue that higher STD and RMSSD observed with ESM techniques may reflect differences in environmental contexts to which depressed individuals are typically more exposed, such as higher levels of bullying and workplace violence and fewer exposure to pleasant events^49–51^.

Partly supporting our second hypothesis, we observed atypical behaviour of the DAN in healthy participants with greater levels of anhedonic depressive symptoms. Attentional difficulties in clinical depression have been well-documented (see Rock et al.^52^ for a meta-analysis). The DAN, involved in externally oriented attention, has shown atypical behaviours both at rest (see Kaiser et al.^53^ for a meta-analysis) and during tasks^54^ in MDD. Unlike Alonso-Martínez et al.^22^, who found lower DAN engagement in subclinical depression at rest, our task required participants’ active engagement through continuous subjective ratings. In fact, in resting-state studies, the absence of any manipulation of attention or emotion hinders the drawing of conclusions about the relationship between atypical network recruitment and self-reported emotional difficulties^29^. Indeed, it is difficult to infer specific patterns of affective processes from unconstrained brain activity^28^.

We also demonstrated, for the first time to our knowledge, that blunted, or less intense, emotional responses (i.e., lower within-subject STD of the subjective ratings) during the task were linked to a higher likelihood of DAN occurrence. Further analyses revealed that MASQ-AD fully mediated the relationship between DAN recruitment and emotional blunting. More specifically, DAN recruitment positively predicted anhedonic depressive symptoms which, in turn, negatively predicted the magnitude, or intensity, of the subjective ratings (i.e., within-subject standard deviation). These findings are consistent with the notions of numbing out and atypical engagement with the external environment previously described by Rottenberg^50^ and the Constructionist theory^55^. Indeed, according to Constructionism, or Theory of Constructed Emotion, the depressed brain keeps making incorrect predictions about the body’s energy needs as if it was chronically predicting and reliving painful events from the past when the metabolic needs were high and costly^56^. In response to these perceived high-energy demands, the depressed brain becomes hyper-focussed on its surroundings, hypervigilant to potential threats, overly prepared to mobilize resources that are not actually needed in the present. In an effort to cut down on perceived “expenditure”, the entire system may eventually shut down, which translates into emotional blunting, reduced motivation, withdrawal from activities and disengagement from the environment^50^.

Exploratory analyses revealed that the relationship between MASQ-AD scores and DAN activity was even more evident while participants listened to neutral songs, particularly following sad music. In more depressed individuals, DAN activity remained elevated during transitions from emotional to neutral music, unlike in less depressed participants. These findings support the Emotion Context Insensitivity (ECI^6^) theory which describes a lack of sensitivity in transitioning between emotional and neutral events in depression. They could reflect a carry-over effect as observed in previous studies where larger levels of lethargy that carried over across days have been found to temporally predict increases in anhedonia^46,47^. This difficulty in recovering from a negative emotional state has also been described in the context of shifting impairments in the transition from negative to neutral information in dysphoric adolescents^57^.

### Limitations

This study has several limitations.

Firstly, “neutral” songs may have affective connotations. Indeed, even though in our study averaged ratings were significantly different between happy and neutral songs, and between sad and neutral songs, previous studies have shown that neutral stimuli may sometimes be interpreted as slightly negative^58^, and this might have affected our findings.

Second, this study did not control for the perceptual properties of each song. Indeed, it is well-known that emotional responses to music may vary with features such as loudness, pitch level or sharpness^59^. Future studies may investigate whether the relationship between the severity of anhedonic depressive symptoms, the subjective ratings and attentional networks recruitment was somehow related to some of the song features. Indeed, this may answer questions about the interactions between attention, perception and emotions.

Thirdly, overall, the happy songs were rated as more familiar by the participants than the sad songs. Some happy songs may have been associated with specific memories in some participants, which might have been reflected in their ratings. However, we did control for familiarity ratings in all analyses. Future studies should consider a post-scan debriefing session with the participants to further investigate potential autobiographical memories at play during the experiment. Additionally, we asked the participants to listen to the pieces of music outside of the scanner once before fMRI data acquisition. Even though this allowed us to successfully collect information about the participants’ familiarity with the different pieces of music, it might also have created a repetition effect, potentially impacting the emotional responses to the music whilst inside the scanner^60^.

In addition, this study was carried out in a healthy sample, which means that, at this stage, we cannot generalise these findings to clinical populations, but this encourages future research to explore dynamic responses to naturalistic paradigms in clinical samples, such as MDD.

Finally, it is worth noting that the background noise generated by the scanner may have impacted on the participants’ emotional responses to the different pieces of music. Indeed, previous research has shown that scanner noise may distort affective brain processes^61^. Future studies would benefit from exploring affective processing in the context of quieter MRI sequences such as Looping Star^62^.

## Conclusion

In this study, we explored behavioural and brain network dynamics as a function of anhedonic depressive symptoms severity in healthy adults during an emotionally provocative music listening task. Our findings highlight an increased occurrence of the Dorsal Attention Network (DAN) in participants with higher levels of anhedonic depressive symptoms, which was associated with a blunted emotional response to both happy and sad songs. In particular, anhedonia mediated the relationship between the occurrence of the DAN and emotional variability metrics. Indeed, increased occurrence of the DAN predicted higher levels of anhedonia which, in turn, predicted blunted emotional responses to both happy and sad pieces of music (i.e., lower within-subject standard deviation). Furthermore, this elevated recruitment of the Dorsal Attention Network during emotional pieces of music carried over into subsequent affectively neutral music in participants with higher anhedonia. Future research should explore whether these findings could be generalised to a clinical population (i.e., major depressive disorder).

### Supplementary Information

Supplementary Information.

## Data Availability

The datasets generated during the current study are available from the corresponding author on reasonable request.
